# Combining venetoclax and azacytidine with T-cell bispecific antibodies for treatment of acute myeloid leukemia: a preclinical assessment

**DOI:** 10.1038/s41375-023-02127-0

**Published:** 2024-01-11

**Authors:** Gerulf Hänel, Anne Schönle, Anne-Sophie Neumann, Daniel Nixdorf, Nora Philipp, Monika Sponheimer, Alexandra Leutbecher, Alica-Joana Emhardt, Giulia Magno, Veit Bücklein, Jan Eckmann, Diana Dunshee, Vesna Kramar, Koorosh Korfi, Sara Colombetti, Pablo Umaña, Christian Klein, Marion Subklewe

**Affiliations:** 1 Department of Medicine III, LMU University Hospital, LMU Munich, Munich, Germany; 2 Laboratory for Translational Cancer Immunology, Gene Center, LMU Munich, Munich, Germany; 3 Roche Pharma Research & Early Development, Roche Innovation Center Zurich, Schlieren, Switzerland; 4 Roche Pharma Research & Early Development, Roche Innovation Center Munich, Penzberg, Germany; 5 Genentech Inc., South San Francisco, CA USA; 6 German Cancer Consortium (DKTK) and German Cancer Research Center (DKFZ), Heidelberg, Germany

**Keywords:** Preclinical research, Translational research, Acute myeloid leukaemia, Cancer immunotherapy

## To the Editor

The development of Venetoclax (Ven) and Azacytidine (Aza) as treatment option in Acute Myeloid Leukemia (AML) has been a breakthrough especially for patients unfit for intensive chemotherapy (IC). Ven/Aza induced complete remission (CR) in 36.7% of newly diagnosed elderly AML patients [1]. However, long term response rates remain poor with a median event-free survival of 9.8 months and a median overall survival of 14.1 months [1], due to persistence of measurable residual disease (MRD) that drives relapse. Novel therapeutic strategies are therefore urgently needed. Data from B-cell malignancies have demonstrated that T-cell based immunotherapy platforms, e.g. T-cell bispecific antibodies (TCBs) or chimeric antigen receptor (CAR) engineered T cells, are able to induce long-term remission and even cure in this patient cohort. Treatment with Blinatumomab in B-cell precursor acute lymphoblastic leukemia resulted in significant elimination of MRD in low-burden disease [2]. Therefore, utility of immunotherapies such as TCBs post Ven/Aza could be envisaged to eradicate the MRD pool in AML and to improve survival rates in this patient population. This concept is further supported by preclinical data that demonstrated a Venetoclax induced increase in reactive oxygen-species (ROS) production in T cells, which resulted in enhanced T-cell effector function [3]. In addition, Azacytidine increased the susceptibility of AML cells for T-cell mediated lysis [3]. Multiple early phase clinical trials with TCBs targeting CD123 (Flotetuzumab and Vibecotamab) or the intracellular tumor antigen Wilms tumor 1 (WT1) presented on HLA-A*02 are currently underway in AML [4–6]. In the current report, we investigate the immune modulating impact of Ven/Aza on the efficacy of a WT1-targeted TCB in AML in vitro and in vivo.

**Fig. 1:**
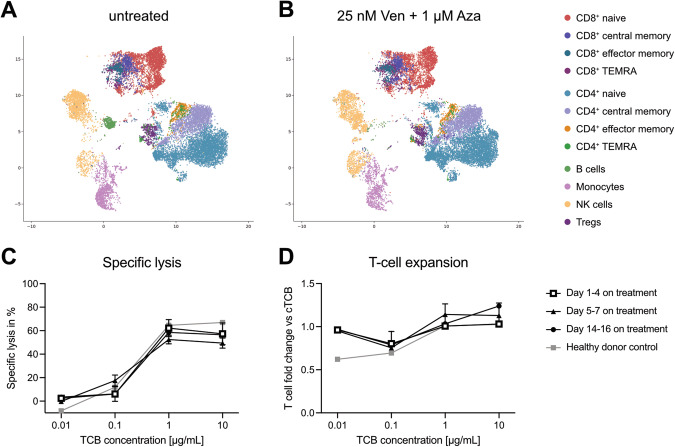
Ven/Aza has limited impact on the T-cell compartment. **A** High dimensional clustering by Cytolution of HD PBMCs (**A**) left untreated or (**B**) treated with 25 nM Ven and 1 µM Aza for three days in vitro (*n* = 3). **C** Specific lysis of OCI-AML3 cells and (**D**) T-cell expansion in cytotoxicity assays with WT1-TCB and T cells from Ven/Aza-treated patients during their first cycle of treatment (*n* = 3 per time point). HD T cells were included as control (*n* = 3). Dots represent mean ± SEM.

To this end, we analyzed the PBMC composition of three healthy donors (HD) in vitro by flow cytometry after three days of treatment with 25 nM Ven and 1 µM Aza, reflecting patient serum concentrations [7, 8], and compared to an untreated control. High dimensional clustering showed no changes in clustering of NK cells and monocytes but revealed a depletion of B cells (Fig. 1A, B). Interestingly, it has recently been shown that bone marrow inflammation in AML is associated with an enrichment of atypical B cells likely conveying suppressive function [9]. Importantly, the clustering of the T-cell compartment remained largely unaffected in our analysis with the exception of CD8^+^ naïve T cells that were partially depleted in response to Ven/Aza (Fig. 1A, B). Ten-fold higher concentrations of Ven further enhanced the reduction of CD8^+^ naïve T cells and led to an overall reduction in viable T cells. A more detailed analysis revealed that the cytoreductive effect in this experiment was only mediated by Ven but not Aza (Supplementary Fig. 1). Taken together, these results confirm previous observations on distinct Ven-sensitivity of lymphocytes [10–12].

Next, we studied primary patient samples derived from Ven/Aza- treated patients. T cells were extracted from peripheral blood on days 1–4, 5–7 and 14–16 post Ven/Aza treatment. We first evaluated the WT1-TCB mediated cytotoxicity against OCI-AML3 cells in a co-culture with the extracted T cells and observed that WT1-TCB consistently and dose-dependently induced target cell lysis (mean specific lysis with 1 µg/mL WT1-TCB: d1-4: 62.2 ± 7.3%; d5-7: 52.5 ± 3.7%; d14-16: 58.5 ± 5.6%; ±SEM; all n = 3; Fig. 1C). Importantly, results showed low inter-patient variability and reflected data obtained from HD T cells. In line with these findings, a dose-dependent T-cell expansion was observed throughout the longitudinal monitoring (Fig. 1D). Taken together, the data indicate that Ven/Aza does not change T-cell function in AML patients during the first two weeks of treatment.

**Fig. 2:**
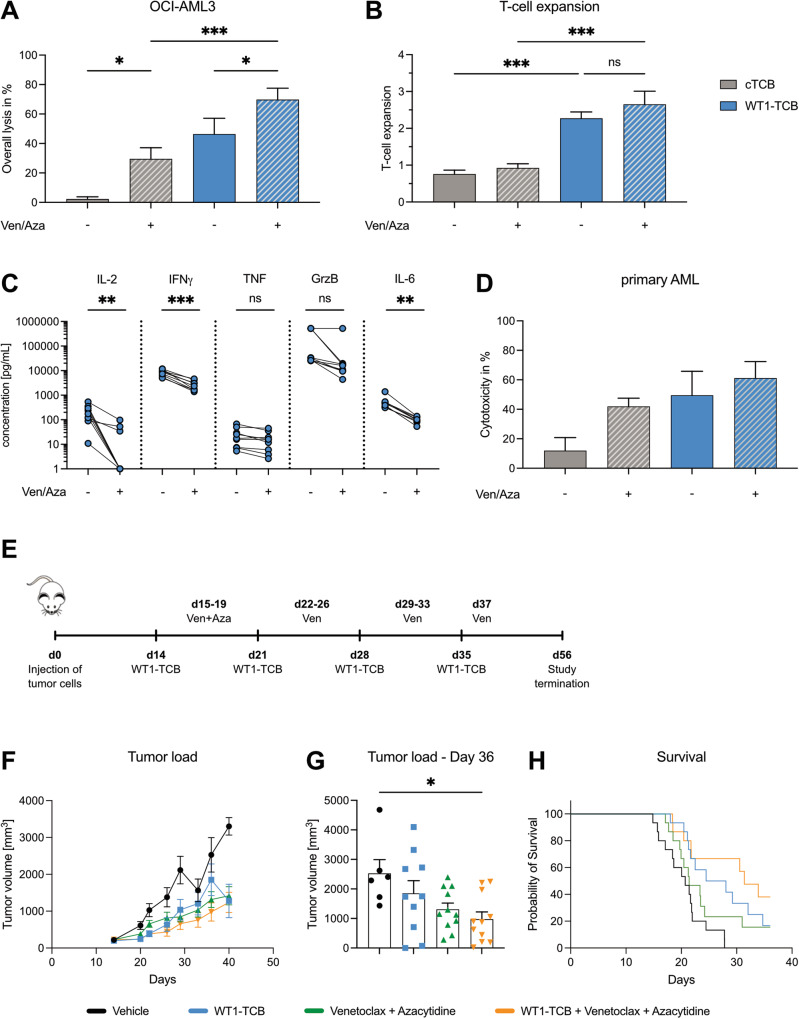
Ven/Aza augments WT1-TCB mediated lysis in vitro and in vivo. **A** Lysis of OCI-AML3 cells and (**B**) T-cell expansion compared to day 0 in cytotoxicity assays with HD T cells mediated by 1 µg/mL cTCB and WT1-TCB after six days in the presence or absence of 25 nM Ven and 1 µM Aza. Bars represent the mean ± SEM (*n* = 9). Statistical analysis: One-way ANOVA with Tukey post hoc test, *p < 0.05, ***p < 0.001; (**C**) cytokine secretion after six days in cytotoxicity assays with OCI-AML3 cells and HD T cells. Statistical analysis: paired *t* test, **p < 0.01, ***p < 0.001; ns, not significant. **D** Lysis of primary AML cells in cytotoxicity assays with HD T cells mediated by cTCB and WT1-TCB after six days in the presence or absence of Ven/Aza. Bars represent the mean ± SEM (*n* = 9). **E** Experimental set-up for in vivo testing of WT1-TCB in combination with Ven/Aza in humanized NSG mice bearing SKM1 tumor. **F**, **G** Tumor load of SKM1 cells over time and on day 36, and (**H**) overall survival of humanized mice treated with WT1-TCB and Ven/Aza (*n* = 15 per treatment group). Bars represent mea*n* ± SEM. Statistical analysis: Dunn’s test, *p < 0.01.

Next, we investigated whether Ven/Aza sensitized AML cells to TCB-mediated lysis. First, we tested the combination of Ven/Aza and WT1-TCB in vitro in cocultures with T cells from nine healthy donors and OCI-AML3 (Fig. 2) or SKM1 cells (Supplementary Fig. 2A, B) for six days in the presence of 1 µg/mL WT1-TCB, 25 nM Ven and 1 µM Aza mimicking concentrations reported for AML patients. A control TCB (cTCB) recognizing a nontumor target antigen was included as control. Ven/Aza mediated cytotoxicity of OCI-AML3 cells was significantly increased in combination with the WT1-TCB from 29.5 ± 7.6% to 69.8 ± 7.8% (*p* = 0.0004; ±SEM; *n* = 9) (Fig. 2A). Moreover, WT1-TCB led to a significant increase in T-cell proliferation in the presence of Ven/Aza (mean fold expansion compared to day 0: 2.7 ± 0.4) (Fig. 2B). Importantly, this was comparable to monotherapy with WT1-TCB (2.3 ± 0.2), whereas Ven/Aza and cTCB treatments did not lead to a T-cell expansion (0.9 ± 0.1 and 0.8 ± 0.1, respectively) (Fig. 2B). Furthermore, we observed lower levels of secreted proinflammatory cytokines IFNγ (*p* < 0.0001), IL-6 (*p* = 0.004), and TNF (*p* = 0.056), as well as secretion of Granzyme B (*p* = 0.125) and IL-2 (*p* = 0.004) in the Ven/Aza and WT1-TCB combination treatment compared to WT1-TCB monotherapy (Fig. 2C).

We have furthermore validated synergism between Ven/Aza and WT1-TCB in cytotoxicity experiments with OCI-AML3 cells and T cells isolated from three AML patients obtained after their first cycle of Ven/Aza. Again, the combinatorial approach increased the lysis compared to monotherapy with Ven/Aza or WT1-TCB (Supplementary Fig. 2C).

Analysis of four primary AML samples further validated our findings. The combination of Ven/Aza and WT1-TCB led to the highest cytotoxicity in cocultures with healthy donor T cells over seven days. While Ven/Aza and WT1-TCB already achieved 40 – 50% lysis, the combinatorial approach enhanced cytotoxicity up to 61.2 ± 11.2% (*n* = 4; Fig. 2D). Similar observations were made using a CD33 targeting TCB with AML cell lines and primary AML cells showing that enhanced tumor cell killing by the combination of Ven/Aza and TCBs is tumor target independent (Supplementary Fig. 2D–F).

Lastly, we applied a humanized HLA-A*02^+^ NSG mouse model bearing SKM1 tumors with 15 animals per treatment group to evaluate the combinatorial effect of Ven/Aza and TCB in vivo. Mice received daily treatments with 1 mg/kg Aza (days 15–19) and 50 mg/kg Ven (days 15–37, except for weekends) and weekly injections with the WT1-TCB (0.05 mg/kg) upon reaching an average tumor size of 198 mm^3^ on day 14 (Fig. 2E). Monotherapies with either WT1-TCB or Ven/Aza inhibited tumor growth (day 36: 1850 ± 432 mm^3^ and 1316 ± 213 mm^3^, respectively), compared to the corresponding vehicle control (2528 ± 468 mm^3^). However, the combination of WT1-TCB and Ven/Aza resulted in significant anti-tumor activity in terms of reduced tumor growth (978 ± 247 mm^3^; *p* = 0.0023) but also prolongation of the median survival (*p* = 0.0034) compared to the vehicle control on day 36 (Fig. 2F–H). Furthermore, treatment with Ven/Aza but not WT1-TCB resulted in a decreased body weight compared to the vehicle control, which was not further increased by the combinatorial treatment (Supplementary Fig. 3). Other apparent signs for clinical toxicities were not observed, indicating no synergistic side effects of Ven/Aza and WT1-TCB.

Taken together, our data provide a first rationale for combining Ven/Aza and TCBs as demonstrated by the significantly increased anti-tumor activity observed in in vitro and in vivo assays. Moreover, we also observed that synergies between Ven/Aza and TCBs are likely independent of the specific tumor target, based on our observations with a CD33-TCB. A challenge in the application of TCBs is the risk for higher-grade cytokine release syndrome which can be mitigated by step-up dosing as shown in trials [13]. Notably, through combination of Ven/Aza and WT1-TCB we observed a reduced secretion of the proinflammatory cytokines IFNγ and IL-6 compared to TCB alone. Therefore, the combination of these drugs might even increase the TCB tolerability, albeit this needs further pre-clinical investigation. Furthermore, the optimal scheduling of Ven/Aza and TCBs whether in parallel or sequential, to increase patients’ benefit/risk ratio requires further evaluation. Overall, our data support the combination of TCBs with Ven/Aza, the current gold standard for unfit and high-risk AML patients, as potentially more efficacious and less toxic anti-leukemic therapy. Clearly, further research is needed to dissect the impact of Ven/Aza on the immune contexture of AML and to identify the most effective treatment strategy.

### Supplementary information

Supplementary Figures

Supplementary Materials and Methods

## Data Availability

All data generated and analyzed during the current study are available from the corresponding author on reasonable request.
