# Supplementary material for: Combining venetoclax and azacytidine with T-cell bispecific antibodies for treatment of acute myeloid leukemia: a preclinical assessment
Source: Leukemia. 2024 Jan 11;38(2):398–402. doi: 10.1038/s41375-023-02127-0 (PMC10844082; doi:10.1038/s41375-023-02127-0)
Supplement: Supplementary file 1 — Supplementary Figures [file 41375_2023_2127_MOESM1_ESM.pdf]

Supplementary Figures

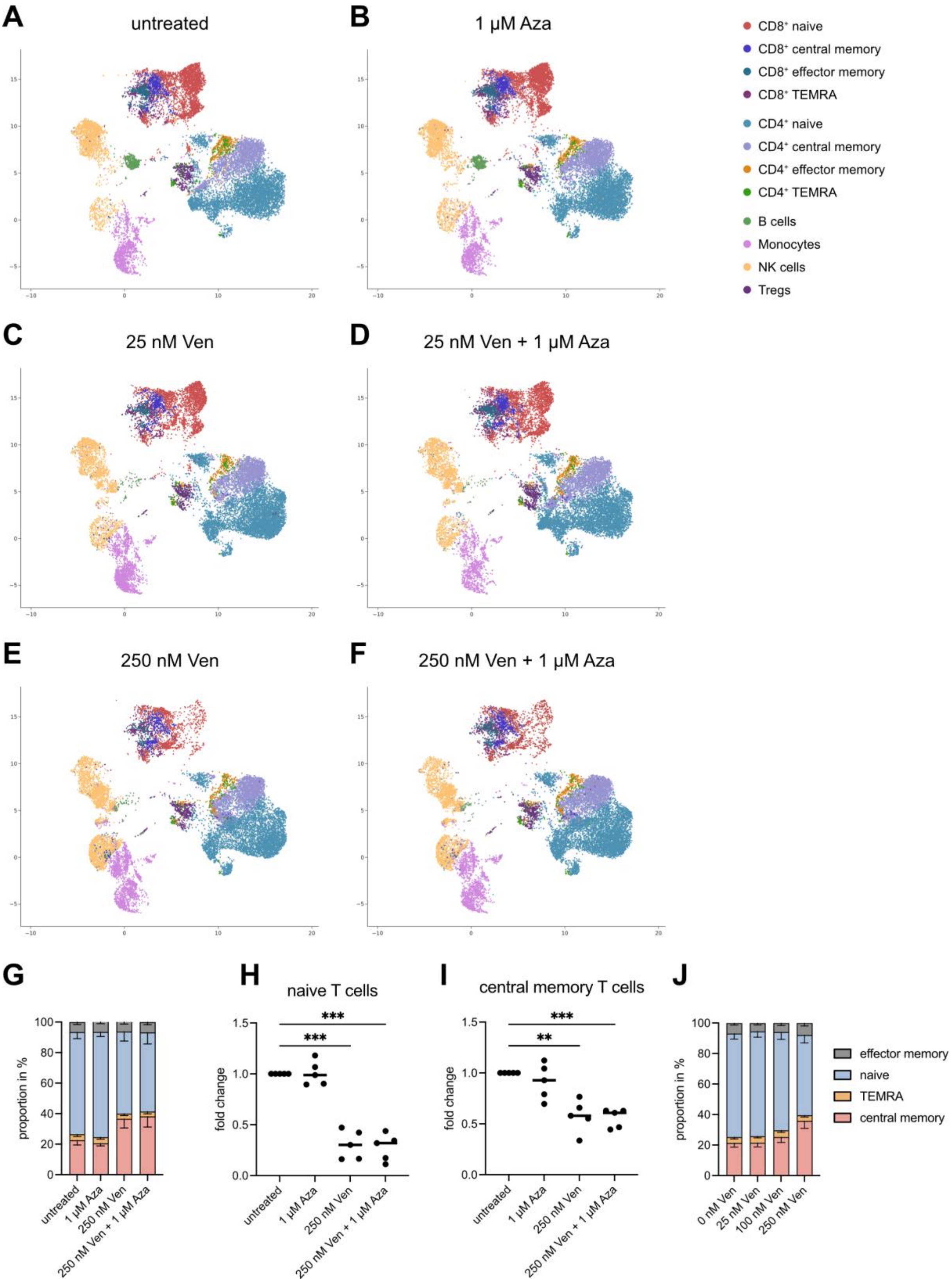

Supplementary Figure 1. Ven/Aza has limited impact on the T-cell compartment. High dimensional clustering by Cytolution of HD PBMCs (A) left untreated, or treated for three days with (B) 1 µM Aza (C) 25 nM Ven (D) 25 nM Ven and 1 µM Aza (E) 250 nM and (F) 250 nM Ven and 1 µM Aza (all n=3). (G) Subset distribution in purified HD T cells after three days treatment with Ven/Aza. Fold change in viable naïve (H) and central memory (I) HD T cells after three days treatment with Ven/Aza relative to untreated controls (all n=5). (J) Subset distribution after three days in purified HD T cells in response to Ven-dose titration (n=6). Data represents the mean±SEM. Statistical analysis: One-way ANOVA with Dunett's post hoc test, **p<0.01, ***p<0.001

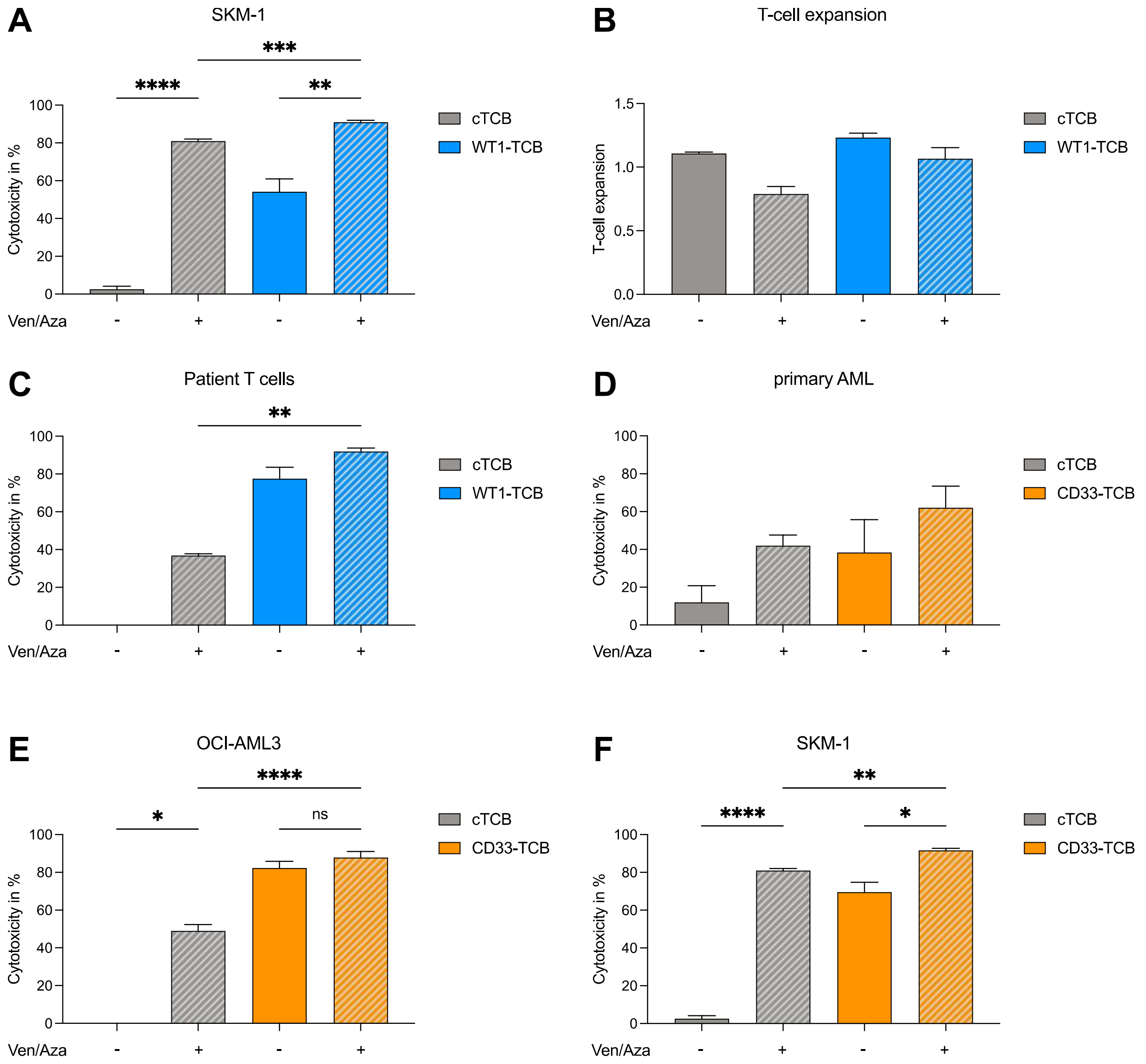

Supplementary Figure 2. The combination of Ven/Aza and TCBs increases cytotoxicity. (A) Lysis of SKM-1 cells and (B) T-cell expansion compared to day 0 in cytotoxicity assays with HD T cells mediated by cTCB and WT1-TCB after three days in the presence or absence of Ven/Aza. (n=3-6). (C) Lysis of OCI-AML3 cells in cytotoxicity assays with T cells from AML patients isolated after their first cycle of Ven/Aza (n=3). Lysis of (D) primary AML cells (n=4), (E) OCI-AML3 cells (n=3) and (F) SKM-1 cells (n=6) in cytotoxicity assays with HD T cells mediated by cTCB and CD33-TCB in the presence or absence of Ven/Aza. Bars represent the mean ± SEM. Statistical analysis: One-way ANOVA with Tukey post hoc test, *$p<0.05$, **$p<0.01$, ***$p<0.001$, ****$p<0.0001$

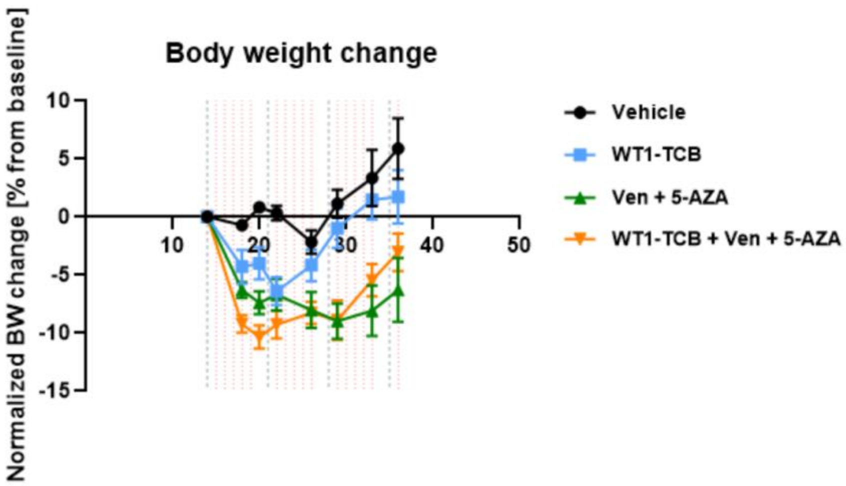

Supplementary Figure 3. Body weight changes in the in vivo experiment evaluating the combination of Ven/Aza and WT1-TCB.

Body weight changes are calculated relative to the start of treatment (*n*=15 per treatment group).
