# Supplementary material for: Combining venetoclax and azacytidine with T-cell bispecific antibodies for treatment of acute myeloid leukemia: a preclinical assessment
Source: Leukemia. 2024 Jan 11;38(2):398–402. doi: 10.1038/s41375-023-02127-0 (PMC10844082; doi:10.1038/s41375-023-02127-0)
Supplement: Supplementary file 2 — Supplementary Materials and Methods [file 41375_2023_2127_MOESM2_ESM.pdf]

Supplementary Materials and Methods

Human samples

Peripheral blood (PB) samples were collected from healthy donors (HDs) and AML patients during their first cycle of Ven/Aza on days 1-4, 5-7 and 14-16 after written informed consent in accordance with the Declaration of Helsinki and approval was granted by the Institutional Review Board of the Ludwig-Maximilians-Universität (Munich, Germany).

Cell lines

Cell lines OCI-AML3 and SKM1 were obtained from the Deutsche Sammlung von Mikroorganismen und Zellkulturen (DSMZ) and were monthly tested for mycoplasma contamination and authenticated by STR profiling in August 2023.

In vitro immune phenotyping in response to Ven/Aza

PMBCs of three healthy donors were resuspended at 1x10^6 cells/mL in R10 medium [RPMI-1640 (PAN-Biotech, Aidenbach, Germany) supplemented with 10% fetal calf serum and penicillin-streptomycin-glutamine (both Life Technologies, Eugene, OR)] and treated with 1 µM Aza (added daily) and 25-250 nM Ven (both Selleckchem, Houston, TX). Cells were subsequently stained for CD3 (clone: UCHT1, catalogue number #612965, BD Biosciences, San Diego, CA), CD14 (61D3, #11-0149-42, Invitrogen, Carlsbad, CA), CD19 (REA675, #130-113-649, Miltenyi Biotec, Bergisch Gladbach, Germany), CD4 (RPA-T4, #300512), CD8 (SK1, #344714), CD25 (BC96, #302646), CD45 (HI30, #368514), CD45RA (HI100, #304108), CD56 (5.1H11, #362506), CD127 (A019D5, #351310), CD197 (G043H7, #353214), HLA-DR (L243, #307642) and Zombie UV Fixable Viability Kit (all Biolegend, San Diego, CA). Cells were subsequently assessed by flow cytometry (CytoFLEX LX, Beckman Coulter, Krefeld, Germany) and data was analyzed using Cytolution (Cytolytics, Tübingen, Germany).

Alternatively, T cells of six healthy donors were isolated using EasySep Human T Cell Isolation Kit (Stemcell, Köln, Germany) according to the manufacturer's instruction. Cells were cultivated in R10 in the presence of 1 µM Aza (added daily) and 25-250 nM Ven for three days. Cells were subsequently stained for CD3 (clone: HIT3a, #300328), CD4 (OKT4, #317408), CD8 (SK1, #344714), CD45RA (HI100, #304130), CD197 (G043H7, #353204) (all Biolegend) and Live/Dead Fixable Aqua Dead Cell Stain Kit Zombie UV Fixable Viability Kit (Invitrogen) and

assessed by flow cytometry (CytoFLEX S, Beckman Coulter). Data was analyzed by FlowJo v10.9 (BD, Ashland, OR) and Prism v10.0.0 (GraphPad Software, San Diego, CA).

Monitoring of T-cell function in AML patients receiving Ven/Aza

T cells were isolated at different time points from peripheral blood of AML patients receiving their first cycle of Ven/Aza using EasySep Human T Cell Isolation Kit (Stemcell). T cells were co-cultured with OCI-AML3 cells at an E:T ratio of 1:2 in the presence of 0.01-10 µg/mL WT1-TCB. Specific lysis of target cells and T-cell expansion was assessed after 3 days relative to a control TCB. Three patient samples per time point were analyzed.

Cytotoxicity assays in combination with Ven/Aza

Primary AML cells were thawed and pre-cultivated on a feeder layer of irradiated murine MS5 stromal cells in a twelve-well plate for 3 days as described before.[1] Healthy donor T cells were co-cultured for 3-6 days with OCI-AML3, SKM-1 or primary AML cells at an E:T ratio of 1:2 in the presence of 25 nM Ven, 1 µM Aza. WT1-TCB was added at a concentration of 1 µg/mL for OCI-AML3 and primary AML cells, whereas 0.01 µg/mL was used for SKM-1 cells. A non-tumor-targeting control TCB (cTCB) was included as control, as well as co-cultures with TCB only. Cytotoxicity assays with primary AML cells were performed on a layer of MS-5 cells. Overall lysis was assessed by flow cytometry on day 6 and calculated relative to an untreated control. T-cell expansion was calculated relative to day 0 based on absolute T-cell counts. Cell culture supernatants were analyzed on day 6 for cytokine secretion using LEGENDplex Human CD8/NK panel according to the manufacturer's instruction.

***In vivo* study with humanized mice**

Female 3-week old NSG (NOD/scid/IL-2Rγnull) mice were irradiated (140cGy) and engrafted by intravenous injection of $9x10^4$ $CD34^+$ cord blood cells per mouse at Jackson Laboratories (Sacramento, US). After reaching a human immune infiltrate (hCD45 above 25% in blood), mice were shipped to Roche and maintained for 5 days to get accustomed to the new environment. Afterwards $1x10^7$ WT1-expressing HLA-A*02^+ SKM-1 tumor cells were subcutaneously inoculated in mice aged 16-26 weeks. After 14 days, when tumor size reached an average volume of 198 mm^3, mice were randomized to four non-blinded treatment groups with 15 animals each and comparable distribution of tumor volume and body-weight. Mice were treated with 0.05 mg/kg WT1-TCB, 50 mg/kg Ven and 1 mg/kg Aza or histidine buffer

(vehicle) for four weeks. Sample size was calculated based on a statistical power of 80% and the assumption of normal distributed results.

Mice were maintained under specific pathogen-free condition with daily cycles of 12-hour light/12-hour darkness according to international [Federation for Laboratory Animal Science Associations (FELASA)] and national [Gesellschaft für Versuchstierkunde/Society of Laboratory Animal Science (GV-Solas) and Tierschutzgesetz (TierSchG)] guidelines. The animal facility has been accredited by the Association for Assessment and Accreditation of Laboratory Animal Care (AAALAC). A body weight loss of 20 % was defined as an endpoint criterion, except for a weight drop observed upon 1st injection of immunomodulatory test substances for which a reduction of 25% for a maximum of 7 days is tolerated. The animal study was approved by and done under license from the Government of Upper Bavaria (Regierung von Oberbayern; Approval number: ROB-55.2-2532.Vet_02-20-170).

References

1 Krupka C, Kufer P, Kischel R, Zugmaier G, Bögeholz J, Kohnke T *et al.* CD33 target validation and sustained depletion of AML blasts in long-term cultures by the bispecific T-cell-engaging antibody AMG 330. *Blood* 2014; **123**: 356–365.
